# The neurocognitive impact of loneliness and social networks on social adaptation

**DOI:** 10.1038/s41598-023-38244-0

**Published:** 2023-07-25

**Authors:** Daniel Franco-O’Byrne, Juan Pablo Morales Sepúlveda, Raúl Gonzalez-Gomez, Agustín Ibáñez, Daniela Huepe-Artigas, Cristián Matus, Ruth Manen, Jaime Ayala, Sol Fittipaldi, David Huepe

**Affiliations:** 1 Center for Social and Cognitive Neuroscience (CSCN), School of Psychology, Universidad Adolfo Ibáñez, Diagonal Las Torres 2640, Peñalolén, Santiago de Chile, Chile; 2 University of Sydney Business School, Darlington, Australia; 3 Facultad de Educación Psicología y Familia, Universidad Finis Terrae, Santiago, Chile; 4 Latin American Brain Health Institute (BrainLat), Universidad Adolfo Ibáñez, Santiago, Chile; 5 Cognitive Neuroscience Center, Universidad de San Andrés, Buenos Aires, Argentina; 6 National Scientific and Technical Research Council (CONICET), Buenos Aires, Argentina; 7 Global Brain Health Institute, Trinity college , Dublin, Ireland; 8 Hospital de Carabineros de Chile, Santiago de Chile, Chile; 9 Facultad de Psicología, Universidad Nacional de Córdoba, Córdoba, Argentina

**Keywords:** Cognitive neuroscience, Social behaviour, Social neuroscience, Neuroscience, Amygdala, Prefrontal cortex

## Abstract

Social adaptation arises from the interaction between the individual and the social environment. However, little empirical evidence exists regarding the relationship between social contact and social adaptation. We propose that loneliness and social networks are key factors explaining social adaptation. Sixty-four healthy subjects with no history of psychiatric conditions participated in this study. All participants completed self-report questionnaires about loneliness, social network, and social adaptation. On a separate day, subjects underwent a resting state fMRI recording session. A hierarchical regression model on self-report data revealed that loneliness and social network were negatively and positively associated with social adaptation. Functional connectivity (FC) analysis showed that loneliness was associated with decreased FC between the fronto-amygdalar and fronto-parietal regions. In contrast, the social network was positively associated with FC between the fronto-temporo-parietal network. Finally, an integrative path model examined the combined effects of behavioral and brain predictors of social adaptation. The model revealed that social networks mediated the effects of loneliness on social adaptation. Further, loneliness-related abnormal brain FC (previously shown to be associated with difficulties in cognitive control, emotion regulation, and sociocognitive processes) emerged as the strongest predictor of poor social adaptation. Findings offer insights into the brain indicators of social adaptation and highlight the role of social networks as a buffer against the maladaptive effects of loneliness. These findings can inform interventions aimed at minimizing loneliness and promoting social adaptation and are especially relevant due to the high prevalence of loneliness around the globe. These findings also serve the study of social adaptation since they provide potential neurocognitive factors that could influence social adaptation.

## Introduction

Social adaptation refers to the extent to which individuals are motivated to participate in their various societal roles and interact with others under societal norms and expectations^1,2^. One crucial factor negatively affecting social adaptation is the experience of loneliness, defined as a distressing emotional state resulting from the subject's perception of separateness, regardless of the actual amount of social contact^3,4^. The maladaptive effect of loneliness is associated with an impoverished social network, poor well-being, disruptive social behavior^5^, and abnormal function in neural networks known to be involved in cognitive control, self-regulation, and social cognition^6,7^. Notably, impoverished social networks and associated limitations can explain the relationship between loneliness and decreased social adaptation. Conversely, social adaptation can significantly benefit from support resources from the social network^8,9^. Despite the above, little is known about the effect of loneliness and social networks on social adaptation, and even less is known about the neural aspects underlying such relationships**.** To approach this issue, we analyzed the relationship between multi-domain measures (i.e. self-report, resting-state functional connectivity) of loneliness and social network on social adaptation. A better understanding of social adaptation may constitute valuable input for public policymaking and interventions promoting development.

Social adaptation is intricately related to social engagement^10,11^. Interaction with other members of society and social institutions (i.e. socialization) allows individuals to gain knowledge about the roles, values, rights, and obligations within their society as well as facilitate coping with contextual demands^12–15^. These outcomes of social interaction foster adaptive behaviors within the social environment^13,16–18^. However, despite the recognized importance of social interaction for social adaptation, there is currently a lack of empirical evidence examining the relationship between these constructs. Consequently, the neurophysiological mechanisms underlying this relationship remain unknown.

One factor that can negatively affect social adaptation is the experience of loneliness. The emotional distress because of loneliness is linked to maladaptive outcomes, including poor physical and mental health^19–21^ and diminished well-being^5,22,23^ and limited ability to fulfill societal roles^24^. Loneliness can also affect social adaptation through increased social isolation. Reduced social interaction and participation reflect poor social integration (low social engagement), a core aspect of social adaptation^25^. On the one hand, decreased contact with others limits the beneficial effects of social interactions, such as obtaining social support^23,26–30^. Support resources are necessary to adjust contextual demands successfully^15,31,32^. Thus, limited access to such resources may result in socio-adaptive difficulties. This situation may perpetuate lonely individuals' tendency to experience reduced reward from social stimuli^33^, perpetuating loneliness and social isolation.

Additionally, Loneliness is associated with abnormal functional connectivity (FC) of attention (Dorsal and ventral networks)^34,35^ and cognitive control networks (Cingulo-opercular network and right middle/superior frontal gyrus)^34,36^. These alterations are known to underpin the maladaptive characteristics of loneliness such as reduced social affiliation (and increased aversion), negatively biased cognition, emotion dysregulation (high social pain), and impaired social cognition^23,30,37–41^. The above findings imply that the association between loneliness and social adaptation may be explained by altered patterns of brain connectivity and decreased social interactions.

On the other hand, one crucial factor that may facilitate social adaptation is the social network^8,42^ . The social network provides support through emotional, instrumental, and informational resources that facilitate social adjustment and coping ability while reducing stress^8,43^. People with better and more complex social networks report fewer daily hassles and lower stress^9,44–46^. At the neurocognitive level, these stress-buffering effects are modulated by prefrontal activation and fronto-limbic functional connectivity associated with adequate cognitive function, self-regulation, and social cognition^32,47,48^. Based on the evidence above, having a rich social network and its associated neurocognitive and stress-buffering effects would be expected to be essential for successful social adaptation. Conversely, it is reasonable to think that the socio-adaptive effects of the social network would be debilitated in subjects experiencing loneliness. However, previous research has not directly assessed the link between these factors.

Considering this background, this work aims to test the association between measures of loneliness, social network, and their FC correlates with social adaptation. We also evaluated the mediating role of the social network in the relationship between loneliness and social adaptation. Based on previous research on the adverse effects of loneliness on well-being and social functioning^5,23^ , we expect that loneliness is negatively associated with social adaptation. On the other hand, considering previous accounts of the stress-buffering hypothesis^9,32,49^, we expect that social networks facilitate social adaptation and explain the potential mechanism underlying the association between loneliness and social adaptation. Regarding the resting-state FC predictors of social adaptation, we expect loneliness-related abnormal function in attentional and cognitive control networks to be negatively associated with social adaptation. We also expect that FC correlates of social networks are positively associated with social adaptation. This study is the first to examine the behavioral and neural factors associated with social adaptation. This study also provides empirical evidence that can aid public policy and serve as a basis for interventions promoting social development.

## Materials and methods

### Participants

Based on an a priori power analysis, our integrative path model (see below), which includes 6 predictors, required data from a sample size of 59 subjects to detect a moderate effect size (*F*^2^ = 0.15) with a power of 0.90 and *α* = 0.05. To ensure adequate power, we recruited a sample of 64 subjects between 20 and 73 years old (*M* = 36.88; *SD* = 13.62; female (n = 38). They have an average of 11 years of education (*M* = 11.1, *SD* = 3.09, range 2–20 years of education) and reported no history of psychiatric or neurological conditions. Participants were recruited by accessibility from the general population.

Individual differences in executive function were controlled using the INECO frontal screening^50^. This instrument is sensitive for assessing executive dysfunction (see Supplementary instruments). The mean score for our sample was 20.92 points, just above the 18-point cut-off for the Chilean population^51^.

The Universidad Diego Portales ethics committee approved every procedure of this research. All participants signed an informed consent according to the principles of the Declaration of Helsinki and received a payment as retribution for their collaboration.

### Procedure

Participants were contacted via telephone or social network media and invited to attend the laboratory to complete various scales tapping on social adaptation, loneliness, and social network. fMRI scanning session was carried out on a different day using a Siemens Avanto 1.5 T scanner equipped with a standard head coil.

### Self-report assessment

#### Social adaptation self-administered Scale (SASS)

The SASS^52^ is a 21-item scale that explores social adjustment and motivation. The SASS was initially developed to evaluate social functioning in clinical populations^52^. However, it has been increasingly used to assess social adaptation in non-clinical populations^11,53–55^. The items tap into levels of engagement with the environment (e.g., "*Do you feel able to organize your environment according to your wishes and needs?*"), family relationships (e.g., "*How frequently do you seek contact with your family members?*"), friendships (e.g., "*What value do you attach to the relationship with others?*"), and engagement to work ("*How interested are you in your occupation?*"), among others. Responses are recorded via a 4-point Likert scale (from 0 to 3). The total score ranges from 0 to 60, corresponding to minimal and maximal social adjustment. Scores between 35 and 52 are normal^52^. The instrument was reliable in our sample (α = 0.73).

#### University of California Loneliness Scale (UCLA)

The UCLA^4^ is a widely used measure of the subject's feelings of loneliness and levels of satisfaction with social experiences. For the present study, an abbreviated version of the UCLA was used^56^, comprising the following eight items: (1) "*I can find companionship when I want it*"; (2) *"I feel left out";* (3) "*I feel isolated from others*"; (4) "*I lack companionship*"; (5) "*There is no one I can turn to*"; (6) "*I am unhappy with being so withdrawn";* (7) "*People are around me but not with me*"; (8)* "I am an outgoing person*". Responses were recorded via a 4-point Likert scale, ranging from 0 (never) to 3 (always). The total score is obtained by inverting positive items and summarizing the score of all items. Thus, more significant scores indicate a more pronounced experience of loneliness. UCLA has shown good levels of reliability, as evidenced by an α coefficient of 0.89^4^. We used a shorter 8-item version that showed good reliability levels in our sample (α = 0.85).

#### The revised Lubben Social Network Scale (LSNS-R)

The LSNS-R is a 12-item scale that measures the size and complexity of social relationships^57^ . It consists of two scales, one tapping on kinship/family ties (e.g., "*How many relatives do you see or hear from at least once a month*?"), and other evaluating non-kin / friendship ties (e.g., "*How many friends do you feel close to such that you could call on them for help?*"). Items are rated on a scale from 0 to 5, with 0 indicating the lowest frequency and number of contact with others and 5 indicating the highest frequency/number of contacts with others. The total score is obtained by summarizing all items' scores. The maximum total score is 60, with higher scores reflecting bigger and stronger social ties. The scale shows high reliability in old adults (α = 0.90)^58^ and young populations (α = 0.83)^59^. A similar level of reliability was obtained in our sample with a Cronbach coefficient of α = 0.85.

### Images data collection

Images for this study were obtained from a Siemens Avanto 1.5 T scanner equipped with a standard head coil. We obtained 10-min resting-state fMRI recordings from 61 participants (data from 3 participants was excluded during pre-processing because of the low quality of their recordings). Functional spin-echo volumes were acquired sequentially ascendingly, parallel to the anterior–posterior commissures, covering the whole brain. The following parameters were used: TR = 3.3 s; TE = 50 ms; flip angle = 90°; number of slices = 36, matrix dimension 4 × 64; voxel size = 3 × 3 × 3.75 mm^3^; number of volumes = 190. Participants were instructed to lay still, keep their eyes closed, and not think about anything particular.

### Data analyses

#### Self-report data

Descriptive data analysis for social adaptation, loneliness, social network, and executive functions are displayed in Table 1. We also conducted correlation analyses, including sociodemographic data and variables of interest (see Table 1).

**Table 1:** Correlation matrix including sociodemographic data and variables of interest.

Variable	M	SD	1	2	3	4	5
1. Age	36.88	13.62					
2. Years of education	11.07	3.09	− 0.50** [− 0.67, − 0.27]				
3. SASS	42.27	7.12	− 0.01 [− 0.26, 0.25]	0.07 [− 0.19, 0.32]			
4. UCLA	8.65	5.27	0.08 [− 0.18, 0.33]	0.04 [− 0.22, 0.29]	− 0.56** [− 0.71, − 0.36]		
5. LSNS_R	34.52	10.47	− 0.21 [− 0.47, 0.08]	− 0.04 [− 0.32, 0.25]	0.48** [0.24, 0.67]	− 0.43** [− 0.63, − 0.17]	
6. IFS Total score	20.92	3.80	− 0.30* [− 0.52, − 0.04]	0.45** [0.21, 0.63]	0.03 [− 0.22, 0.28]	0.02 [− 0.23, 0.27]	0.23 [− 0.05, 0.48]

Correlation analysis for our sample of 64 subjects (37 female, 27 male). *M* and *SD* are used to represent mean and standard deviation, respectively. Values in square brackets indicate the 95% confidence interval for each correlation. The confidence interval is a plausible range of population correlations that could have caused the sample correlation (Cumming, 2014). Note that * indicates *p* < 0.05. ** indicates *p* < 0.01. Abbreviations: SASS (social adaptation self-administered scale), UCLA (University of California loneliness scale), LSNS_R (Lubben social network scale).

Behavioral data were analyzed with R studio^60^. We first conducted a hierarchical multiple regression to evaluate the predictive value of loneliness and social network on social adaptation. Hierarchical multiple regression models help evaluate and compare the predictability of groups of independent variables entered at different steps of the analysis^61^. In other words, the main idea of the analysis is to test whether variables entered in subsequent steps have better predictive value than those entered in a former step of the analysis. As for the present analysis, we first specified a base model including our control variables (executive functions, age, education, and gender). These variables did not have any significant effect on social adaptation. In a subsequent step, our measure of loneliness was incorporated into the group of variables. For the last step, we specified a model that included the social network measure (LSNS scores).

### Resting-state fMRI data

#### Pre-processing

First, we discarded the first three volumes of each subject's resting-state recording to ensure that magnetization achieved a steady state. Images were then pre-processed using the Data Processing Assistant for Resting-State fMRI (DPARSF V2.3)^62^. This open-access toolbox generates an automatic pipeline for fMRI analysis. The DPARFS processes the data recruiting the Statistical Parametric Mapping (SPM12) and the Resting-State fMRI Data Analysis Toolkit (REST V.1.7). In line with recommendations^63^, pre-processing included slice-timing correction (using the middle slice of each volume as the reference scan) and realignment to the first scan of the session to correct head movement (SPM functions). To reduce the effect of motion during image acquisition as well as physiological artifacts^64^, we controlled two motion parameters (i.e. Translation, rotation; See Supplementary table 1), CFS, and WM signals (REST V1.7 toolbox). Motion parameters were estimated during realignment, and data from three subjects were discarded due to exceeding the maximum head movement (3 mm and 3°). After discarding the three subjects, the subsample left showed acceptable motion parameters in movements (*M* = 0.05, SD = 0.04) and rotations (*M* = 0.05, SD = 0.03). CFS and WM masks were derived from the tissue segmentation of each subject's T1 scan in native space with SPM12 (after co-registration of each subject's structural image with the functional image). Then, images were normalized to the MNI space using the echo-planar imaging (EPI) template from SPM^65^, smoothed using an 8-mm full-width-at-half-maximum isotropic Gaussian kernel (SPM functions), and bandpass filtered between 0.01 and 0.08 Hz. None of the participants showed movements greater than 3 mm (M = 0.05, SD = 0.04) or rotations higher than 3° (M = 0.05, SD = 0.03).

### Functional connectivity analyses

We explored associations between resting-state functional connectivity data and scores from our predictor variables, loneliness (UCLA scores) and social network (LSNS-R score). First, for each subject, we extracted the mean time course of the BOLD signal in each of the 116 regions of the Automated Anatomical Labelling Atlas (AAL)^66^ by averaging the signal in all voxels comprising each region. Second, we constructed a connectivity matrix for each subject, indicating the strength of association between all pairs of regions (Pearson's correlation coefficient; DPARSF toolbox). Third, we performed a Fisher z-transformation. The resulting functional connectivity (FC) correlation coefficients between all pairs of regions (AAL atlas) were used to perform Spearman's correlations with the scores of each predictor: loneliness (UCLA score) and social network (LSNS-R score). Following procedures from recent research^67–69^, the significance thresholding of neuroimaging results was set to *p* ≤ 0.001 (uncorrected). Less stringent, uncorrected statistical thresholds, as supported by previous research^70–72^, can be valuable in reducing false negative results, particularly in modest sample sizes like ours. This approach allows for the consideration of genuine effects that might have otherwise been disregarded with stricter thresholds.

### Principal component analyses (PCA) of fMRI data

We conducted PCA for each predictor separately to reduce the dimensionality of the FC correlates of the two predictors (loneliness and social network). In the case of loneliness, we analyzed the rho values of the pairs of regions that significantly correlated with UCLA scores (see Supplementary table 2). For social networks, we also analyzed the rho values of the pairs of regions that significantly correlated with LSNS.R scores (see Supplementary table 3). Then, the component that captured the most variance associated with each construct was retained and included in the posterior integrative path analysis.

### Integrative path analysis

We performed a path analysis using the Laavan package^73^ in JASP statistical software to evaluate the combined effect of self-report and brain indicators of social adaptation. This technique tests a theoretical model comprising (a priori) hypothesized relationships between variables. Based on this approach, we constructed a model that integrates self-report and neurophysiological (FC) data as predictors of social adaptation. Specifically, we hypothesize that at the behavioral (self-report) level, loneliness scores are associated with lower performance on the social adaptation scale (SASS). Social network scores will mediate this relationship. These patterns of associations will be replicated at the neurophysiological level, meaning that FC correlated to loneliness will predict low performance in the social adaptation scale. In contrast, FC correlated to social networks will mediate in such relationships. The fit is evaluated by various parameters, including the X^2^ statistic (non-significant), NFI (> 0.95), GFI (> 0.95), CFI (0.95–1.00), RMSEA (< 0.08), IC (≤ 0.05), SRMR (< 0.08).

Path models can be used to extend the multiple regression model by simultaneously analyzing the relationships between the independent and dependent variables^74^ . Based on this approach, we proposed a model incorporating brain variables with purely behavioral ones to generate an integrated model.

### Ethics approval

The Universidad Diego Portales ethics committee approved every procedure of this research. All participants signed an informed consent according to the principles of the Declaration of Helsinki and received a payment as retribution for their collaboration and time**.**

## Results

Correlations for our analyzed variables are displayed in Table 1 below.

### Self-report measures

#### Multiple hierarchical regression: loneliness and social network as predictors of social adaptation

We performed a hierarchical multiple regression to evaluate the predictive value of loneliness and social network on social adaptation (see Table 2). Before the hypothesized model, we conducted the first analysis step by considering sociodemographics and EF as control variables. Results showed that gender (β = 0.13, *p* = 0.41), age (β = 0.04, *p* = 0.81) years of education (β = 0.09, *p* = 0.67) and executive function (β = − 0.04, *p* = 0.82) did not have significant effects over social adaptation. In the second step, after controlling the intervening variables, only loneliness (β = − 0.58, p < 0.001) emerged as a significant predictor of Social adaptation[*F*(1, 38) = 17.82, p < 0.001]. In a third step, the social network was added to the existing predictors; this model revealed that both loneliness (β = − 0.41, p < 0.005) and social network (β = 0.43, p < 0.005) significantly predicted social adaptation. Interestingly, this model shows that the effect of loneliness is still significant after including the social network. After comparing the R^2^_adj._ values for the last two steps, the third step explains 13% more variance associated with social adaptation than the second. Finally, regarding the effects of potentially confounding variables, we confirmed that the associations amongst the variables of interest remained significant after controlling for gender, age, years of education, and EFs.

**Table 2:** Multiple hierarchical regression for loneliness and social network as predictors of social adaptation.

	Demographic			Loneliness			Social network		
	Std. Beta	Standardized CI	*p*	Std. Beta	Standardized CI	*p*	Std. Beta	Standardized CI	*p*
(Intercept)	0.00	− 0.32–0.32	< 0.001*	0.00	− 0.26–0.26	< 0.001*	0.00	− 0.24–0.24	0.002*
Gender	0.13	− 0.19–0.46	0.411	− 0.02	− 0.30–0.26	0.884	− 0.04	− 0.29–0.22	0.783
Age	0.04	− 0.33–0.42	0.818	0.16	− 0.16–0.47	0.325	0.25	− 0.05–0.54	0.099
Years of education	0.09	− 0.33–0.51	0.673	0.12	− 0.23–0.47	0.500	0.26	− 0.07–0.59	0.123
Executive functions	− 0.04	− 0.42–0.33	0.828	0.04	− 0.28–0.35	0.803	− 0.08	− 0.38–0.22	0.596
UCLA				− 0.59	− 0.87 to − 0.31	< 0.001*	− 0.41	− 0.70 to − 0.13	< 0.05*
LSNS							0.43	0.14–0.71	< 0.05*
R^2^/R^2^ adjusted	0.025/− 0.075			0.336/0.249			0.466/0.380		

*Note* The table shows standardized coefficients and p-values for each step comprising the hierarchical multiple regression model. The demographic column shows parameter estimates for our control variables (i.e. sociodemographic data and executive functions). The middle column shows the model evaluating the effects of loneliness on social adaptation after controlling for non-interest variables. After controlling for demographic variables, the final column shows the model evaluating the effects of loneliness and social network on social adaptation. Asterisks indicate significant effects. For more details, refer to Supplementary table 4.

*UCLA* University of California Loneliness Scale, *LSNS* Lubben Social Network scale.

Significant values are in bold.

### Functional connectivity results

**Figure 1:**
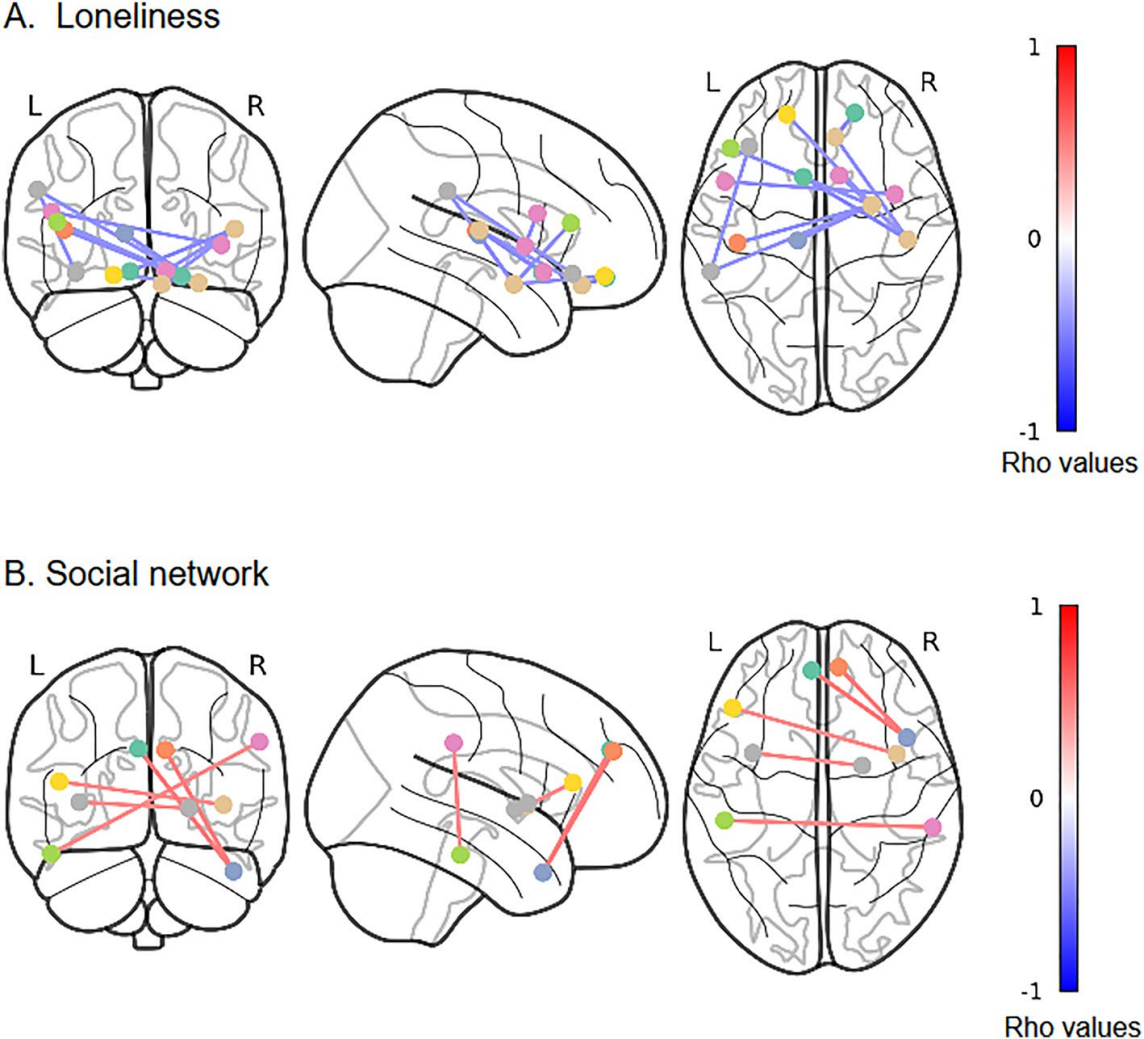
(**A**) Resting-state functional connectivity negatively associated with loneliness (UCLA score), (**B**) Resting-state functional connectivity positively associated with Social networks (LSNS-R score). The figure above shows pairs of areas with a threshold of *p* ≤ 0.001. *Lines connecting the nodes illustrate the valence of the association (between self-reported scores and pairs of areas), with blue lines reflecting decreased connectivity and red lines reflecting increased connectivity. Also, note that the color of the nodes (circles indicating the areas) have no other meaning than to facilitate the differentiation of each region. For more details on the areas depicted in this figure, see Supplementary tables 2 and 3

Significant functional connectivity for loneliness and social network is represented in Fig. 1A and B, respectively (for the list of specific areas involved, please see Supplementary tables 2 and 3). Loneliness was negatively associated with the functional connectivity between the amygdala and frontal regions (i.e. orbitofrontal gyrus, superior and inferior frontal gyrus) and the supramarginal gyrus in the parietal cortex. Fronto-insular connectivity was also negatively correlated to loneliness (see Fig. 1A and Supplementary table 2). The social network was associated with increased functional connectivity between the frontotemporal and temporoparietal areas and connectivity between the insula, frontal areas, and basal ganglia (Fig. 1B and Supplementary table 3).

### Principal component analyses (PCA) on Functional connectivity (FC) data

**Figure 2:**
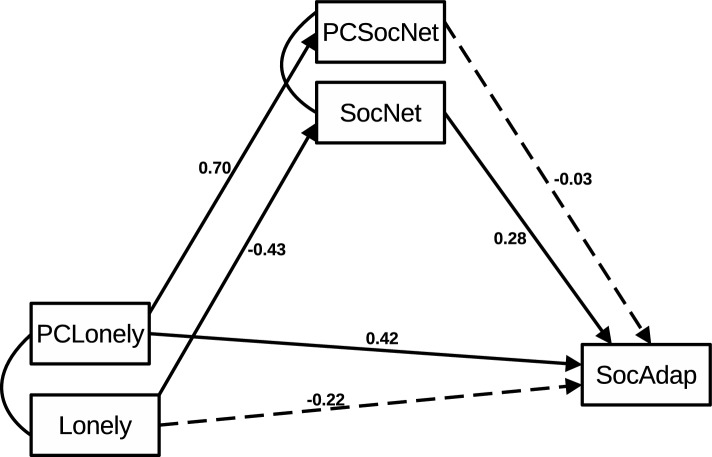
Schematic representation of the integrative path analysis exploring the association between self-reported and FC data with social adaptation. The relationships between the variables are expressed in standardized estimates. Continuous lines represent significant results, while discontinuous lines reflect non-significant effects. The curved lines represent the correlation between two variables (i.e. self-report scores with their FC correlates). Abbreviations: PCLonely: Principal component reflecting functional connectivity negatively associated with loneliness; Lonely: scores in the self-report measure of loneliness (UCLA); PCSocNet: Principal component reflecting functional connectivity positively associated with Social networks; SocNet: scores in the self-report measure of social network (LSNS); SocAdap: scores in the self-report measure of social adaptation (SASS). For more details, see Supplementary Table 5.

To obtain a summarized index of the functional connectivity correlates of each predictor, we conducted PCA and retained the component that captured the most variance. For loneliness, the first component explained 47% of the variance of its functional connectivity. For social networks, the first component explained 70% of the variance associated with its functional connectivity. These components for both loneliness (PCLonely) and social network (PCSocNet) were included in the subsequent Integrative Path analysis (see Fig. 2).

### Integrative path analysis

A graphic account of the model and results can be seen in Fig. 2. Loneliness negatively relates to social networks (β = − 0.430, *p* < 0.001) and does not have effects on social adaptation (β = − 0.226, *p* = 0.098). Social networks, in turn, have a positive relationship with social adaptation (β = 0.284, *p* < 0.05). The PCA of brain connectivity of loneliness (PCLonely) had positive effects on social adaptation (β = 0.428, *p* < *0.05*).

Additionally, PCLonely showed significant positive effects on the PCA of social network (PCSocNet) (β = 0.705, *p* < *0.001*), which in turn had non-significant effects on social adaptation (β = − 0.038, *p* = *0.814*).

Regarding the model's fit, the χ2 test statistic was not significant, suggesting that the model had a good fit (χ^2^ [4, N = 47] = 2.16, *p* = 0.71). Overall, the fit indices were very good; NFI = 0.99 (values above 0.95 is good); GFI = 0.99 (values above 0.95 is good); CFI = 1.00 (values in the range of 0.95–0.99 considered as excellent fit, and a value of 1 considered as exact fit); RMSEA = 0.00, IC = 0.00–0.16 (values ≤ 0.05 considered as good fit); SRMR = 0.04 (a value less than 0.08 is generally considered a good fit).

## Discussion

This study is the first to assess the relationship between loneliness, social network, and their FC correlates with social adaptation. We hypothesized that loneliness and its functional brain connectivity (FC) correlates were negatively associated with social adaptation, while social network and brain correlates would explain such maladaptive effects. Our hierarchical multiple regression indicates that loneliness was associated with low scores on the social adaptation scale. The model also revealed that the effect of loneliness on social adaptation decreased after including the social network measure, which positively affected social adaptation. The model containing both factors as predictors explained the most variance associated with social adaptation.

Loneliness revealed a decreased functional connectivity of the amygdala and fronto-parietal areas. These regions have previously been shown to underpin social pain, emotional disorders, cognitive control, and mentalizing impairment. On the other hand, the social network was positively associated with intrinsic FC between previously reported hubs for global cognitive functioning and self-regulation. However, this FC did not have predictive value over social adaptation. The final integrative path analysis model showed that at the behavioral level (self-reported data), social networks mediated the effects of loneliness over social adaptation. At a neurophysiological level, social adaptation was predicted solely by the resting-state FC correlates of loneliness. These FC correlates of loneliness explained most of the variance associated with social adaptation when considering behavioral and brain factors. These findings offer new insight into behavioral, affective, socio-cognitive, and, more importantly, neurophysiological predictors of social adaptation. Our findings may also serve as an empirical basis for interventions to improve well-being and development.

Previous research has highlighted the maladaptive impact of loneliness on health, cognitive functioning, and social behavior^5,23,41^ and the stress-buffering role of the social network^8,43^. In line with this evidence, our hierarchical regression results suggest that feeling lonely or left out might hinder social adaptation while having a rich social network might facilitate social adaptation. Importantly, two particularities of this model can lead to interesting interpretations. First, the relationship between SA and loneliness or social network remained significant after controlling for each other, indicating that these constructs are differentially associated with social adaptation. Based on the literature cited above, we suggest that the "loneliness-adaptation" link may reflect increased social pain (reduced well-being, and mental health). In contrast, the "social network-adaptation" link reflects enhanced coping capabilities (i.e. mainly due to support resources). The second observation is that introducing the social network measure captures some loneliness-SA variance, suggesting that social networks might explain a mechanism underlying such a relationship.

Decreased contact with others may limit support resources necessary to cope with stress and life demands^8,32,49^, hindering social adaptation. This antecedent is consistent with our finding that social networks mediated the association between loneliness and social adaptation (see Fig. 2). The evidence suggests that loneliness affects social adaptation through isolation from the social group and decreased instances of social support. In this sense, contact with the social network (and obtained social support) may promote social adaption by minimizing emotional distress and fomenting self-regulation and coping^8,43^. These findings highlight previously unaddressed socio-affective and behavioral factors associated with social adaptation.

At the Neurophysiological level, our integrative path model yielded exciting results. Specifically, we found that loneliness-related decreased activity in front-amygdalar and fronto-parietal networks was associated with lower social adaptation scores. Past research shows that the strength of front-amygdalar coupling modulates emotion regulation processes^75^, affective aspects of social perception, and affiliative behaviors^76^. The inability of frontal cortical regions to modulate amygdala reactivity results in emotional dysfunctions, anxious and depressive symptomatology, and isolation exhibited by lonely individuals^41^. In line with the above, our results suggest that decreased front-amygdalar connectivity might indicate poor social adaptation through emotion regulation impairments and poor mental health.

On the other hand, fronto-parietal areas are part of a widely distributed cognitive control network^77^. Cognitive control enables the appropriate selection of thoughts, emotions, and behaviors based on contextual demands^78^. Another aspect associated with fronto-parietal function is the modulation of socio-cognitive abilities (i.e. mentalizing, empathy)^79,80^. Our results suggest that dampened front-amygdalar and fronto-parietal activity in loneliness may hinder social adaptation. This process could be through emotion regulation impairments, inability to cope with daily life stressors, and affected social cognition (i.e. Negative appraisal of social stimuli and poor empathic abilities).

These maladaptive neurocognitive patterns might be explained via lowered oxytocin neuropeptide levels. It is well known that loneliness is associated with attenuated normal oxytocinergic responsiveness, with subjects with higher loneliness having lower oxytocin levels in response to social stimuli^81,82^. This oxytocinergic underproduction could be potentially linked to neurocognitive abnormalities leading to poor social networks and, consequently, maladaptation. For example, research shows that oxytocin neuropeptide modulates emotion regulation by increasing amygdala-prefrontal cortex connectivity^83^. Oxytocin also enhances trust and social approaching by modulating insula reactivity to positive social stimuli^84^.

Regarding sociocognitive impairments, evidence shows that oxytocin weakens negativity bias within regions involved in the theory of mind (temporoparietal junction) and identification of emotional cues in social perception (right fusiform)^85^. The above might offer insights into the potential neuroendocrine mechanisms by which loneliness reduces social networks and affect social adaptation. However, this interpretation is merely speculative, and future studies should directly test the role of oxytocin in the relationship between loneliness, social networks, and social adaptation.

Concerning potential limitations, it is crucial to emphasize that the findings presented in this study are derived from a correlational design. Although there is strong supporting evidence for the proposed relationships, caution should be exercised when inferring causal relationships between variables. On the other hand, we acknowledge that self-report variables may not entirely reflect the construct under investigation. However, according to the theory, such variables showed a pattern of expected relationships.

### Word of caution

Given that this is the first study on this particular topic, we have taken a less stringent approach to explore the association of functional connectivity with self-reported data, as done in previous studies^67–69,86^. However, it is important to note that the significance threshold used in our analysis (p < 0.001, uncorrected) may be considered liberal, which warrants caution when interpreting our findings. To ensure the reliability of the results, future research should aim at replicating our findings using more stringent significance criteria.

## Conclusion

Little has been done to evaluate the aspects that predict and facilitate social adaptation from a socio-affective and neural perspective. This work aimed to predict the effects of socio-affective, cognitive, and brain predictors of social adaptation. Our findings suggest that the negative impact of loneliness on social adaptation involves impairments in social functioning, affective disorders, and decreased functional connectivity in networks associated with cognitive control, self-regulation, and social cognition. However, support from the social network is a critical factor that protects against these maladaptive behavioral and brain patterns and foments adequate social adaptation. Considering this, future research should aim at two primary goals. One is to incorporate measures of the above processes as indicators of social adaptation. The second is to explore further the various contributions of social integration to the social adaptation processes. The findings here reported contributing to the understanding of mechanisms involved in social adaptation. A better understanding of these mechanisms can inform public policymaking and promote social development interventions.

## Supplementary Information

Supplementary Information.

## Data Availability

The data supporting this study's findings are available from the corresponding author upon reasonable request.
