# Supplementary material for: The neurocognitive impact of loneliness and social networks on social adaptation
Source: Sci Rep. 2023 Jul 25;13:12048. doi: 10.1038/s41598-023-38244-0 (PMC10368735; doi:10.1038/s41598-023-38244-0)
Supplement: Supplementary file 1 — Supplementary Information. [file 41598_2023_38244_MOESM1_ESM.docx]

**Supplementary material**

**Supplementary Instruments**

**INECO Frontal Screening (IFS)**

The IFS is a sensitive screening tool to assess executive functioning ^1^. It is composed of 8 sub-tests tapping on 3 executive functions: response inhibition and set-shifting, working memory, and abstraction capacity. The maximum total score is 30, with higher scores indicating better executive functioning. Validation for the Chilean population ^2^ revealed a cut-off: of 18 points). The mean score for our sample was 20.92 points, just above the 18-point cut-off for the Chilean population. As for internal consistency, the IFS is notably good (Cronbach’s alpha = 0.90) ^2^. The executive function measure was included as a control variable in the posterior analysis.

**Supplementary tables**

**Supplementary table 1. Sample’s motion parameters in fMRI**

|  | *M* | SD |
| --- | --- | --- |
| Rotation | 0.04711285 | 0.04099567 |
| Translation | 0.5431649 | 0.03830877 |

The mean and standard deviation for the sample movement during the recording of resting-state fMRI. Rotation movements are expressed in millimeters and translation movements are expressed in degree

**Supplementary table 2. Functional connectivity is negatively associated with loneliness (UCLA score)**

|  | **Spearman’s rho** | ***p*-value** |
| --- | --- | --- |
| Superior frontal gyrus R - Gyrus Rectus R | -0.45 | <.001 |
| Olfactory Cortex L - Heschl’s gyrus R | -0.43 | <.001 |
| Amygdala R - Heschl’s gyrus L | -0.42 | <.001 |
| Amygdala R - Thalamus L | -0.42 | <.001 |
| Inferior frontal gyrus opercular L - Insula R | -0.41 | =.001 |
| Olfactory cortex R - Heschl’s gyrus R | -0.40 | =.001 |
| Inferior frontal gyrus triangular L - Amygdala R | -0.40 | =.001 |
| Superior frontal gyrus orbital L - Amygdala R | -0.40 | =.001 |
| Gyrus rectus R - Heschl´s gyrus R | -0.40 | =.001 |
| Amygdala R - Supramarginal gyrus L | -0.39 | =.001 |
| Inferior frontal gyrus orbital L - Supramarginal L | -0.39 | =.001 |

The first column shows the pair of areas (from the AAL atlas) that are functionally connected. The second column shows the strength and direction of the association between each pair of areas and the score on the self-report scale of loneliness (UCLA). The last column contains the estimated p-value for each association

**Supplementary table 3. Functional connectivity is positively associated with social network (LSNS-R score)**

| **Regions** | **Spearman’s rho** | ***p*-value** |
| --- | --- | --- |
| Superior frontal gyrus L – Medial temporal pole R | 0.50 | <.001 |
| Superior frontal gyrus medial R - Medial temporal pole R | 0.49 | <.001 |
| Supramarginal R - Inferior temporal pole L | 0.45 | =.001 |
| Inferior frontal gyrus triangular L – Insula R | 0.44 | =.001 |
| Insula L - Pallidum R | 0.44 | =.001 |

The first column shows the pair of areas (from the AAL atlas) that are functionally connected. The second column shows the strength and direction of the association between each pair of areas and the score in the self-report scale of social network (LSNS-R). The last column contains the estimated p-value for each association

**Supplementary table 4. Hierarchical multiple regression output showing raw estimates.**

| **Demographics** | | | | **Loneliness** | | | **Social network** | | |
| --- | --- | --- | --- | --- | --- | --- | --- | --- | --- |
|  | *estimates* | *CI* | *p* | *estimates* | *CI* | *p* | *estimates* | *CI* | *p* |
| (Intercept) | 40.26 | 22.23 –52.29 | <0.001* | 41.81 | 26.71 –56.91 | <0.001* | 27.93 | 11.31 – 44.55 | 0.002* |
| Gender | 2.02 | -2.89 – 6.93 | 0.411 | -0.31 | -4.57 – 3.95 | 0.884 | -0.53 | -4.41 – 3.34 | 0.783 |
| Age | 0.02 | -0.18 – 0.23 | 0.818 | 0.09 | -0.09 – 0.26 | 0.325 | 0.13 | -0.03 – 0.29 | 0.099 |
| Years of education | 0.20 | -0.76 – 1.17 | 0.673 | 0.27 | -0.53 – 1.08 | 0.500 | 0.60 | -0.17 – 1.36 | 0.123 |
| Executive functions | -0.08 | -0.77 – 0.62 | 0.828 | 0.07 | -0.51 – 0.66 | 0.803 | -0.15 | -0.70 – 0.41 | 0.596 |
| Loneliness |  |  |  | -0.78 | -1.16 – -0.41 | <0.001* | -0.55 | -0.92 – -0.17 | 0.005* |
| Social network |  |  |  |  |  |  | 0.33 | 0.11 – 0.54 | 0.005* |
| R^2^/R^2^ adjusted | 0.025/ 0.075 |  |  | 0.336/ 0.249 |  |  | 0.466/ 0.380 |  |  |

The table shows unstandardized coefficients and p-values for each of the steps comprising the hierarchical multiple regression model. The demographic column shows parameter estimates for our control variables (i.e. sociodemographic data and executive functions). The middle column showed the model evaluating the effects of loneliness on social adaptation after controlling for non-interest variables. The final column shows the model evaluating the effects of loneliness and social network on social adaptation after controlling for non-interest variables. Asterisks indicate significant effects

**Supplementary table 5. Integrative path analysis output**

| **label** | **est** | **se** | **z** | **p** | **CI**  **(lower)** | **CI**  **(upper)** | **Std**  **(lv)** | **Std**  **(all)** | **Std**  **(nox)** |
| --- | --- | --- | --- | --- | --- | --- | --- | --- | --- |
| **Social adaptation ~ Social network** | 0.202 | 0.091 | 2.222 | 0.026 | 0.024 | 0.380 | 0.202 | 0.284 | 0.284 |
| **Social adaptation ~ Loneliness** | -0.297 | 0.179 | -1.653 | 0.098 | -0.648 | 0.055 | -0.297 | -0.226 | -0.226 |
| **Social adaptation ~ PCSocNet** | -0.153 | 0.651 | -0.235 | 0.814 | -1.430 | 1.123 | -0.153 | -0.038 | -0.038 |
| **Social adaptation ~ PCLonel** | 1.277 | 0.508 | 2.516 | 0.012 | 0.282 | 2.272 | 1.277 | 0.428 | 0.428 |
| **Social network ~ Loneliness** | -0.791 | 0.224 | -3.532 | < .001 | -1.230 | -0.352 | -0.791 | -0.430 | -0.430 |
| **PCSocNet ~ PCLonel** | 0.526 | 0.071 | 7.390 | < .001 | 0.387 | 0.666 | 0.526 | 0.705 | 0.705 |
| **Loneliness ~~ PCLonel** | -7.712 | 2.263 | -3.408 | < .001 | -12.148 | -3.276 | -7.712 | -0.573 | -0.573 |
| **Social network ~~ PCSocNet** | 5.352 | 1.894 | 2.826 | 0.005 | 1.640 | 9.065 | 5.352 | 0.452 | 0.452 |

**Supplementary Results**

**Principal component analyses (PCA) of fMRI data**

For loneliness, we analyzed the rho values of the pairs of regions that significantly correlated with UCLA scores at the threshold significant level of *p* ≤ 0.001 (see *supplementary table 4*). The first component explained 47% of the variance and was used in subsequent analyses.

For social networks, we also analyzed the rho values of the pairs of regions that significantly correlated with LSNS.R scores at the threshold significant level of *p* ≤ 0.001 (see *supplementary table 5*). The first component explained 70% of the variance and was used in subsequent analyses.

**References**

1. Torralva, Roca, Gleichgerrcht, López, M. INECO Frontal Screening (IFS): a brief, sensitive, and specific tool to assess executive functions in dementia. *J Int Neuropsychol Soc* **15**, 777–786 (2009).

2. Ihnen, J., Antivilo, A., Muñoz-Neira, C. & Slachevsky, A. Chilean version of the INECO Frontal Screening (IFS-Ch): Psychometric properties and diagnostic accuracy. *Dement. Neuropsychol.* (2013). doi:10.1590/s1980-57642013dn70100007
